# Upper-Limb Electromyogram Classification of Reaching-to-Grasping Tasks Based on Convolutional Neural Networks for Control of a Prosthetic Hand

**DOI:** 10.3389/fnins.2021.733359

**Published:** 2021-10-12

**Authors:** Keun-Tae Kim, Sangsoo Park, Tae-Hyun Lim, Song Joo Lee

**Affiliations:** ^1^Center for Bionics, Biomedical Research Institute, Korea Institute of Science and Technology, Seoul, South Korea; ^2^College of Medicine, Korea University, Seoul, South Korea; ^3^Department of Physical Therapy, Graduate School, Korea University, Seoul, South Korea; ^4^Division of Bio-Medical Science and Technology, KIST School, Korea University of Science and Technology, Seoul, South Korea

**Keywords:** myoelectric interfaces, reaching-to-grasping tasks, Southampton Hand Assessment Procedure, convolutional neural network, electromyogram

## Abstract

In recent years, myoelectric interfaces using surface electromyogram (EMG) signals have been developed for assisting people with physical disabilities. Especially, in the myoelectric interfaces for robotic hands or arms, decoding the user’s upper-limb movement intentions is cardinal to properly control the prosthesis. However, because previous experiments were implemented with only healthy subjects, the possibility of classifying reaching-to-grasping based on the EMG signals from the residual limb without the below-elbow muscles was not investigated yet. Therefore, we aimed to investigate the possibility of classifying reaching-to-grasping tasks using the EMG from the upper arm and upper body without considering wrist muscles for prosthetic users. In our study, seven healthy subjects, one *trans*-radial amputee, and one wrist amputee were participated and performed 10 repeatable 12 reaching-to-grasping tasks based on the Southampton Hand Assessment Procedure (SHAP) with 12 different weighted (light and heavy) objects. The acquired EMG was processed using the principal component analysis (PCA) and convolutional neural network (CNN) to decode the tasks. The PCA–CNN method showed that the average accuracies of the healthy subjects were 69.4 ± 11.4%, using only the EMG signals by the upper arm and upper body. The result with the PCA–CNN method showed 8% significantly higher accuracies than the result with the widely used time domain and auto-regressive-support vector machine (TDAR–SVM) method as 61.6 ± 13.7%. However, in the cases of the amputees, the PCA–CNN showed slightly lower performance. In addition, in the aspects of assistant daily living, because grip force is also important when grasping an object after reaching, the possibility of classifying the two light and heavy objects in each reaching-to-grasping task was also investigated. Consequently, the PCA–CNN method showed higher accuracy at 70.1 ± 9.8%. Based on our results, the PCA–CNN method can help to improve the performance of classifying reaching-to-grasping tasks without wrist EMG signals. Our findings and decoding method can be implemented to further develop a practical human–machine interface using EMG signals.

## Introduction

Nowadays, the myoelectric interfaces based on electromyogram (EMG) have been developed for supporting the daily living of amputees. Especially, due to its ease of use and non-invasiveness for supporting daily living by interacting with external devices, the myoelectric interfaces have become a useful technology (Hargrove et al., 2007; Castellini et al., 2009). Examples of the myoelectric interfaces include prosthetic arm and hand (Peerdeman et al., 2011; Scheme and Englehart, 2011; Fougner et al., 2012; Chowdhury et al., 2013; Ison and Artemiadis, 2014; Kim et al., 2014), teleoperation robotic devices (Fukuda et al., 2003; Shenoy et al., 2008; Wolf et al., 2013), and gaming interfaces (Saponas et al., 2010).

**FIGURE 1:**
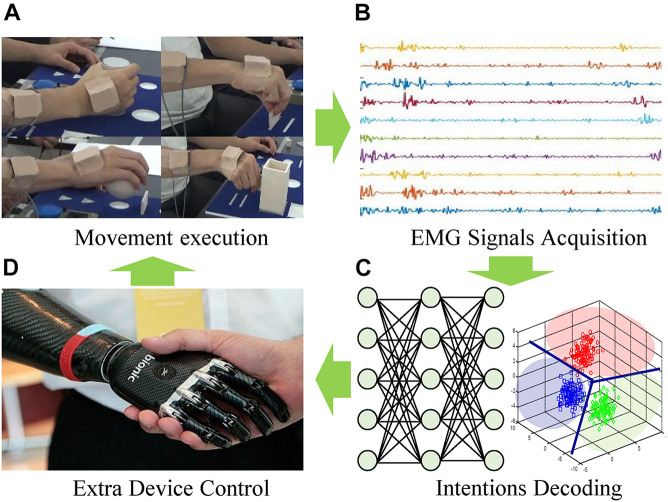
Overview of myoelectric interfaces. **(A)** Movement excution. **(B)** EMG signals acquisition. **(C)** Intentions decoding. **(D)** Extra device control.

The overview of the representative myoelectric interface for an external device is illustrated in Figure 1. The EMG signals are acquired from the user’s movement execution resulting from the user’s movement intentions. Then, suitable features are extracted and classified *via* advanced pattern recognition and machine learning algorithms. So far, the time and frequency domains-based various characteristics, as well as numerous optimal classifiers, have been studied in detail to improve the performance of the classification of the movement intent with varying degrees of success (Zardoshti-Kermani et al., 1995; Chu et al., 2007; Phinyomark et al., 2013; Ameri et al., 2014; Park et al., 2015; Kim et al., 2019). Finally, the classified user’s intentions are decoded as control commands for interfacing with external devices.

The human’s upper-limb movement for grasping an object is a complex task, and various experiments and researches have been implemented (Fligge et al., 2013). Especially, the differentiated control of all fingers is difficult to achieve because of the high dimensionality of the degrees of freedom (DOFs) of the hand (Batzianoulis et al., 2017). The human hand has 21 DOFs controlled by 29 muscles (Jones and Lederman, 2006). This means that humans can control the large number of DOFs of their hands skillfully *via* a multidimensional reduction in the central nervous system-controlled variables (Batzianoulis et al., 2017). This multidimensional reduction, i.e., substantial reduction of DOFs during grasping objects, may be accomplished through the use of postural synergies (Santello et al., 1998) associated with many hand postures during grasping objects (Batzianoulis et al., 2017). In this context, several works studied a mapping between hand postures and upper-limb EMG signals (Smith et al., 2008; Dalley et al., 2011; Ouyang et al., 2013; Sapsanis et al., 2013) as a strategy to control a large number of the hand’s movement. These studies investigated grasping when the hand had already reached the final configuration. In these studies, subjects were asked to perform the appropriate grasp while the upper-arm stalled. However, in the case of reaching-to-grasping movement, the configuration of the hand changes simultaneously with the arm’s motion, including the upper arm and forearm, because the hand’s pre-shape is defined before reaching their final configuration following the characteristics of the object, such as the shape, weight, etc. For example, humeral rotation is closely related to object orientation (Marotta et al., 2003), and transportation time and peak speed can be affected by object size at the same time (Marteniuk et al., 1987).

Recently, Brochier et al. (2004) validated that the upper arm and shoulder muscles in monkeys contain valuable information to discriminate grip types and object locations. In this case, the long flexor muscles and the intrinsic muscles appear to be involved only while the phase related to the generation of force, whereas all other muscles of the upper and lower extremities were active during all different phases and showed a significant interdependency between the actions performed during reach-to-grasp. This means that, for the classification of different grasping tasks, it may not require to record activities at more distal muscles, such as the wrist muscles.

In this context, Martelloni et al. (2008) showed that the development of a pattern recognition method could discriminate EMG signals recorded at the proximal muscles (the deltoideus pars anterior, trapezius pars ascendens, pectoralis major pars clavicularis, triceps brachii caput longum, biceps brachii caput longum, extensor carpi radialis, and flexor carpi radialis) during grasping different objects placed within the different positions. The experiments were implemented with healthy subjects, and the subjects performed reaching and grasping for the three types of objects (a tennis ball, a tin, and a key), which were placed in three different table positions (Martelloni et al., 2008). As a result, the experimental results showed the ability to distinguish between different handles (i.e., palmar, side, or pinch grip) with objects based on EMG signals (Martelloni et al., 2008). However, due to the experiments were implemented with only healthy subjects, an additional experiment is required to be implemented for considering the various types of users, such as the transradial amputees.

In the cases of most *trans*-radial amputees, because they have the EMG signals from the lower-arm muscles such as the flexor carpi radialis, and extensor carpi radialis, classification strategies for the healthy subjects can be applied. Indeed, numerous studies have been conducted to classify upper-limb movements in transradial patients using the muscles, which close to the wrist, activation patterns (Chu et al., 2007; Batzianoulis et al., 2017; Kim et al., 2019). However, there are cases of transradial amputees who do not have the muscles below-elbow or have difficulty controlling the muscles due to deficits in muscle strength and control that could happen due to accidents at very young ages. For these cases, a few studies were conducted for classifying the finger, wrist, and elbow movements using upper-limb muscles, such as triceps and biceps (Young et al., 2012; Jarrassé et al., 2016; Gaudet et al., 2018) until now. Young et al. (2012) showed the possibility of classifying the hand open/close and wrist flexion/extension using the EMG signals with EMG signals based on biceps and triceps. Furthermore, Jarrassé et al. (2016) showed that the individual phantom finger movements (flexion/extension), as well as the wrist and elbow, can be classified using only EMG signals extracted at the upper arm. Moreover, Gaudet et al. (2018) showed the possibility of classifying between the upper-limb phantom movements (elbow flexion/extension, forearm pronation/supination, etc.) and a no-movement, using the EMG from site exclusively on the amputee stump. In the studies mentioned earlier (Young et al., 2012; Jarrassé et al., 2016; Gaudet et al., 2018), the time-domain features, such as the mean absolute value (MAV), slope sign changes (SSC), zero-crossing (ZC), waveform length (WL), root mean square, autoregressive coefficients (AR), etc., were extracted and classified using the linear discriminant analysis (Young et al., 2012; Jarrassé et al., 2016) and multi-layer perceptron (Gaudet et al., 2018).

In the studies mentioned earlier (Young et al., 2012; Jarrassé et al., 2016; Gaudet et al., 2018), simple upper-limb movements, such as flexion or extension, were classified to investigate the possibility. However, in the aspects of the myoelectric interface for control the prosthetic hand, further study to investigate the possibility of the complex upper-limb movements, such as reaching-to-grasping, will be required to assist the daily living of the upper amputees who may not have or have weak below-elbow muscle activities. To the author’s best knowledge, the possibility of classifying reaching-to-grasping based on the EMG signals from the residual limb without the below-elbow muscles was not investigated yet.

Moreover, in the aspects of assisting daily living, the handgrip force is significant to grasp various types of objects. However, relatively few applications of pattern recognition using force classification are found (Khan et al., 2021). A recent study by Jitaree and Phukpattaranont (2019) classified different levels of forces during pinch grasp for EMG signals within healthy subjects. Al-Timemy et al. (2015) classified three broadly divided levels of grip forces using EMG signals from the *trans*-radial amputees. However, in these studies, the EMG signals from the forearm were mainly used, and the muscles of the residual limb above the elbow of patients were not considered yet.

Thus, this study aims to investigate the possibility of classifying the reaching-to-grasping tasks using the EMG signals from upper-body muscles above the elbow for considering the upper-limb amputees who may not have or have weak below-elbow muscle activities. We also investigated the effect of object weights on the possibility of classifying the reaching-to-grasping tasks. The main hypothesis is that if the EMG can be activated at proximal muscles during reaching-to-grasping tasks, the pattern recognition or machine learning techniques may classify reaching-to-grasping tasks using the EMG signals from the upper body.

The main contributions of this study can be summarized as follows. First, we investigated the possibility of classifying the six reaching-to-grasping tasks using the EMG signals from six upper-body muscles above the elbow. Second, the convolutional neural network (CNN) was applied to data processing for classifying the tasks, and the performances were compared with the traditional feature, such as MAV, SSC, ZC, and WL, root mean square, etc. Last, we also investigated the possibility of classifying reaching-to-grasping tasks for different weighted (light and heavy) objects. Consequently, we tried to investigate the possibility of developing a novel myoelectric interface that can control prosthetics based on recognizing the complex upper-limb movements. The feasibility of the algorithm was tested from the *trans*-radial amputee and one wrist amputee, who have weak muscle activities, and then use the six muscles for classifying reaching-to-grasping tasks.

In the remainder of this article, *Materials and Methods* presents the data collection and details the proposed method. *Experimental Results* presents the results of comparisons among the previous methods and the proposed method. Then, the results are discussed in *Discussion*. Finally, our conclusion and future work are presented in *Conclusion*.

## Materials and Methods

### Subjects and Prosthetic Hand

Seven healthy subjects, one *trans*-radial amputee, and one wrist amputee participated in our experiments (Table 1). All healthy subjects were male and right-handed. All patients wore the same type of myoelectric hand prosthesis, bebionic hand (Ottobock, Germany), and had experienced in hand motion control. Their sockets were well-fitted before the experiment. The bebionic hand controlled the hand flexion by activating the wrist flexor and hand extension by activating the wrist extensor. Thereby, EMG activities of wrist extensor and flexor were not able to collect from this study, as those signals were not able to be accessed for data collection. Furthermore, by changing control modes through clicking a button on the back of the bebionic hand and controlling the flexor and extensor activities, there were eight different control modes that subjects could use, namely, tripod grip, power grip, active index, pinch group, key grip, finger point, column, and mouse grip. The prosthetic hand that subjects used in our study did not include the wrist rotation, but the thumb could be manually moved in two modes allowing changes in thumb location either in the neutral position and in the flexion position for an opposite grip. All subjects provided written informed consent approved by the Institutional Review Board of the Korea Institute of Science and Technology before the experiment.

**TABLE 1:** Subjects’ information.

**Info.**	**Healthy (S1–S7)**	**Amputee (S8)**	**Amputee (S9)**
Age (years)	25.3 ± 3.3	25	24
Height (cm)	174.5 ± 8.2	178.5	178.5
Mass (kg)	73.7 ± 11.5	77.7	101.1
Dominant side	Right	–	Right
Tested side	Right	Right	Right
Amputation level	–	Wrist	*Trans*-radial
Amputation years	–	24	5

### Electromyogram Data Acquisition

**FIGURE 2:**
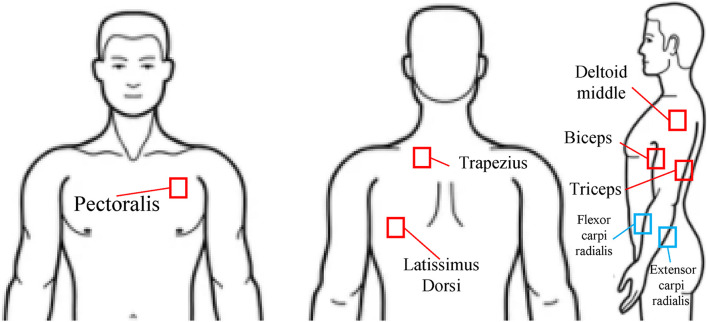
EMG electrode positions (pectoralis, trapezius, latissimus dorsi, deltoid middle, biceps, triceps, flexor carpi radialis, and extensor carpi radialis).

For the data acquisition, the surface electrodes (Bagnoli, Delsys, Inc., Natick, MA, United States) were placed on six major upper body muscles. Three electrodes were attached at the upper arm (deltoid middle, biceps, and triceps), and three electrodes were attached at the trunk muscles (latissimus dorsi, trapezius, and pectoralis). In the cases of the healthy subjects, the two additional electrodes were attached at the forearm (flexor carpi radialis and extensor carpi radialis) (Figure 2). Because of the socket, those forearm muscle activities from the subjects wearing the prosthetic hand were not able to obtain. The EMG signals were recorded by a customized LabVIEW program (National Instruments, Austin, TX, United States). The sampling rate was 1,000 Hz.

### Southampton Hand Assessment Procedure Protocol

For reaching-to-grasping tasks, we used the Southampton Hand Assessment Procedures (SHAP) related to the abstract object (Kyberd et al., 2009). The abstract objects are shaped as six standard prehensile patterns (tip, lateral, tripod, spherical, power, and extension) (Napier, 1956; Kamakura et al., 1980; MacKenzie and Iberall, 1994) and of two different (light/heavy) weights to test the subject’s ability to form more powerful grips (Table 2). For the SHAP, self-timed tasks (the subject starting and stopping the timer with the tested hand) and a form-board were used for our experiment. This aims to eliminate the assessor’s error (Mcsp and Dipcot, 2003).

**TABLE 2:** Objects’ weights.

**Grips**	**Light (g)**	**Heavy (g)**
Spherical	26	530
Tripod	1	21
Power	18	540
Lateral	17	222
Tip	1	69
Extension	2	138

**FIGURE 3:**
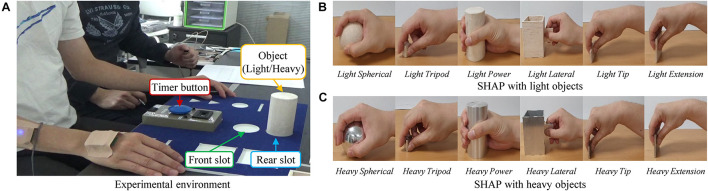
Experimental environments with SHAP protocol. **(A)** Environments for SHAP protocol. **(B)** Grip types for SHAP with light objects. **(C)** Grip types for SHAP with heavy objects.

In our experiments, the subject was seated at a table with the shoulders relaxed; the elbow joint angles were 90° (Figure 3). The form board was positioned at the front of the table with the object to be moved with the subject’s midline. The board was moved for each task. The timer was slotted on the center of the form board. Each task was conducted with the subject’s pushing the timer button for starting and reaching the object, moving the object to the front slot from the rear slot of the form board, and pushing the timer button for ending. The subjects performed 10 times in each task repeatedly (six reaching-to-grasping tasks for the six standard prehensile patterns with two different weights).

### Data Processing

Recently, CNN has arisen as one of the significant approaches in machine learning. Following the advances of computing power achieved through the development of graphics processors, CNN has now been applied to user intention recognition (MacKenzie and Iberall, 1994; Mcsp and Dipcot, 2003; Atzori et al., 2016). In the previous studies, principal component analysis (PCA) and CNN showed better performance for classifying the EMG signals from hand movements. Therefore, the PCA was used to extract spectrogram features from the EMG signals, and simple CNN was designed and used as a classifier in our experiments.

For a fair comparison between subjects and across trials, the acquired EMG data of each channel were normalized using the difference between maximum and minimum values for each muscle across all trials. The normalized data were segmented for time normalization from 0% (initiation of the task) to 100% of the trial completion based on triceps EMG activities as a movement onset, as the triceps muscle was activated when the button of each SHAP task was pushed. The grasping timing was 40% for healthy subjects and 49% for prosthetic hand users on average. Because the classification was needed to control the prosthetic hand just before grasping, 0–40% (healthy subject, S1–S7) and 0–49% (*trans*-radial and wrist amputees, S8 and S9) from the front of each segmented data were used for the data processing to investigate EMG classification algorithm during the duration of reaching-to-grasp.

The segmented data were sectioned into 400 samples with a 50-sample moving window. Then, each segment of each channel was processed independently for the extraction of the spectrogram and normalization. The spectrogram was extracted from each segment using a fast Fourier transform and a Hamming window (Zhai et al., 2017). Therefore, the spectrogram was derived from 129 different frequencies with three-time bins. Also, the first 95 frequencies were used (Zhai et al., 2017). The size of the spectrogram was 95 × 3 × 6 or 8 (frequency × time bins × channels). The spectrograms were converted into a range of 0–1 through maximum–minimum normalization. Then, the normalized spectrograms were vectorized at the channel for improving computational efficiency and performance. The PCA was applied to reduce the dimensionality while maintaining the useful information from the EMG signals. The PCA was only calculated on all the segments across all the classes in the training set. Because the 100–500 principal components (PCs) were enough to perform well (Zhai et al., 2016), only the results of the 25 PCs in each channel were used as input data. Consequently, each spectrogram was reduced to a dimension 25 × 6 or 8 (PCs × channels).

**FIGURE 4:**
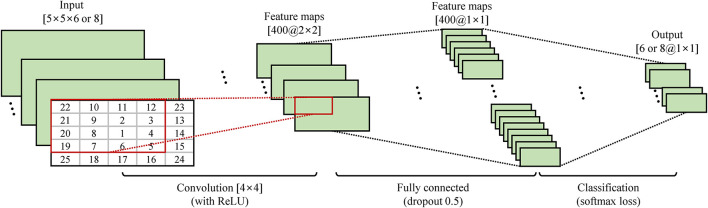
Architecture for PCA–CNN method. Twenty-five PCs were extracted via PCA and reshaped and rearranged into a 2D matrix. Then, two-dimensional matrix was convolved with 400 filters of size 4 × 4 and 2 × 2 and fully connected for classification.

For CNN input, the resulting matrices (25 PCs) were first rearranged to 5 × 5 matrices for each channel (Zhai et al., 2017). To optimize the use of CNN, the PCs were rearranged where the score of the most significant (Figure 4) was at the center. In this way, the most important PCs can be captured by the most convolution filters, which maximize their contribution to the network.

The proposed simple CNN architecture is shown in Figure 4. CNN consisted of four layers. The first convolutional layer was a with 400 filters of size 4 × 4. The second layer was a rectified linear unit, which is a non-linear activation function. To avoid the vanishing gradient problem, the rectified linear unit was used. The third layer contained one fully connected with a size of 400 (dropout rate of 0.5). The fourth layer was a softmax loss for classification. The softmax loss layer calculated the cost function *via* the normalized exponential function. It also printed out the probabilities of all types of movement considered in the current forecast. After several tests, the CNN was trained based on a stochastic gradient descent with a learning rate of 0.001, and the batch size was fixed at 32 and a momentum of 0.9. A MatConvNet, an open-source MATLAB toolbox, was used to implement the CNN structure (Vedaldi and Lenc, 2015). Consequently, the PCA–CNN method was followed by the previous study (Zhai et al., 2017) except for some parameters, such as the number of channels (previous study: 12, our study: 6 or 8), filters (previous study: 800, our study: 400), and iteration (previous study: 300, our study: 100).

### Performance Evaluation

To validate the effectiveness of the PCA–CNN method, we conducted an offline simulation using 10-fold cross-validation with the previous time domain and auto-regressive (TDAR) feature extraction and the support vector machine (SVM) (Englehart and Hudgins, 2003; Park et al., 2015). For the TDAR feature, the MAV, ZC, SSC, WL, and AR were extracted and concatenated. Then, the SVM was used as a classifier. In 10-fold cross-validation, the segmented EMG data were divided into 10-fold randomly without any overlap. Also, ninefold was used for training, and the remaining onefold was used for testing. The testing fold was changed in chronological order. Consequently, the results were averaged to measure the accuracy in the PCA–CNN and TDAR–SVM methods. To obtain a better quantitative comparison between the PCA–CNN and TDAR–SVM methods, we performed the Kolmogorov–Smirnov test to check the normality of data first, and then, we performed the two-way repeated-measures analysis of variance (ANOVA) with *post hoc* tests. Furthermore, Bonferroni correction was also done for multiple comparisons. The *p*-value < 0.05 indicates a statistical significance.

## Experimental Results

### Classification of Reaching-to-Grasping Tasks

**FIGURE 5:**
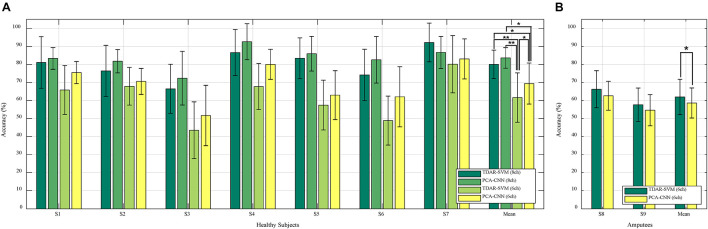
Results of classifying reaching-to-grasping tasks with 10-fold cross-validation for **(A)** healthy subjects and **(B)** *trans*-radial and wrist amputees (* and ** means *p*-value < 0.05 and 0.01, respectively).

Figure 5 presents the 10-fold cross-validation results of the TDAR–SVM and PCA–CNN method within the **(A)** healthy subjects and **(B)** *trans*-radial and wrist amputees for classifying reaching-to-grasping tasks. In Figure 5A, the PCA–CNN method shows higher accuracy than the TDAR–SVM method, using the EMG from six electrodes, as well as eight electrodes, in all subjects. As the averaged accuracies of the healthy subjects were 80.1 ± 7.8 and 83.6 ± 5.7%, 61.6 ± 13.7, and 69.4 ± 11.4%. In general, the PCA–CNN method showed approximately 3 and 8% higher accuracies than the TDAR–SVM method in the eight and six electrodes, respectively.

Furthermore, the two-way repeated-measures ANOVA showed a significant effect for classifier factor [*F*(1,6) = 14.33, *p* = 0.009], a significant effect for the number of channels factors [*F*(1,6) = 40.33, *p* = 0.001], and a significant interaction [*F*(1,6) = 8.84, *p* = 0.025)]. As the interaction effect was significant, we performed multiple pairwise comparisons with Bonferroni *p*-value adjustment. The significant differences were revealed between the “TDAR–SVM (8ch)” and the “TDAR–SVM (6ch)” (*t* = 7.22, *p* = 0.002), between the “PCA–CNN (8ch)” and the “TDAR–SVM (6ch)” (*t* = –6.02, *p* = 0.006), between the “PCA–CNN (8ch)” and the “PCA–CNN (6ch)” (*t* = 5.12, *p* = 0.013), between the “TDAR–SVM (8ch)” and the “PCA–CNN (6ch)” (*t* = 5.10, *p* = 0.013), and between the “TDAR–SVM (6ch)” and the “PCA–CNN (6ch)” (*t* = –4.86, *p* = 0.017). There was no significant difference between the “TDAR–SVM (8ch)” and the “PCA–CNN (8ch)” method (*t* = –2.08, *p* = 0.497). Consequently, although both algorithms performed better when the number of electrodes was eight, statistically better classification ability of the PCA–CNN method was observed only when six electrodes were used for the classification.

On the other hand, in Figure 5B, the TDAR–SVM method showed better performance than the PCA–CNN method within *trans*-radial and wrist amputees as the averaged accuracies of 62.0 ± 9.8% and 58.6 ± 8.3%. Following the statistical analysis, a significant difference was also revealed between the “TDAR–SVM (6ch)” and the “PCA–CNN (6ch)” methods, as *p* < 0.05.

### Confusion Matrices for Reaching-To-Grasping Task

**FIGURE 6:**
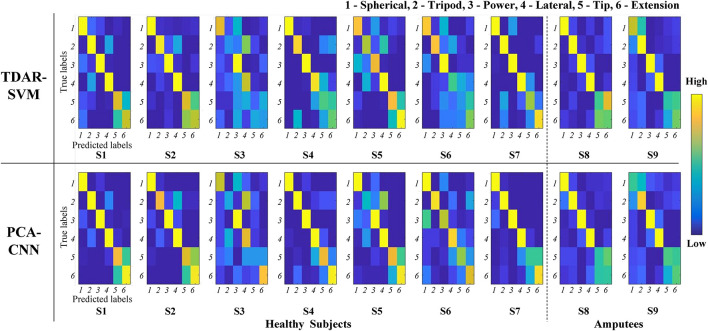
Confusion matrices of classification result from 10-fold cross-validation for healthy subjects and *trans*-radial and wrist amputees. *Y*-axis means true labels, and *x*-axis means predicted labels from classifier. Numbers at true and predicted labels mean reaching-to-grasping tasks (1, spherical; 2, tripod; 3, power; 4, lateral; 5, tip; and 6, extension).

To make it clear in regard to the comparison of the TDAR–SVM and PCA–CNN method for the healthy subjects and *trans*-radial and wrist amputees, we calculated the confusion matrix within the six EMG channels. Figure 6 shows that the calculated confusion matrix from the 10-fold cross-validation for all subjects. In Figure 6, the *y*-axis means the true labels, and the *x*-axis means the predicted labels from the classifier (the TDAR–SVM and PCA–CNN, respectively) in each subject. The color of the confusion matrix presents the classification accuracy of each task. In general, the PCA–NN method showed higher accuracies in each reaching-to-grasping task. Especially, in the case of S4, the PCA–CNN method was shown higher accuracy at all tasks. In the case of S2, S4, and S6, the PCA–CNN method was also shown higher accuracy at the 5 (tip) and 6 (extension) tasks. However, in the cases of the 5 and 6 tasks for *trans*-radial and wrist amputees (S8 and S9), both the PCA–CNN and the TDAR–SVM method showed very lower accuracy.

### Classification of the Different Weights Within the Same Task

**FIGURE 7:**
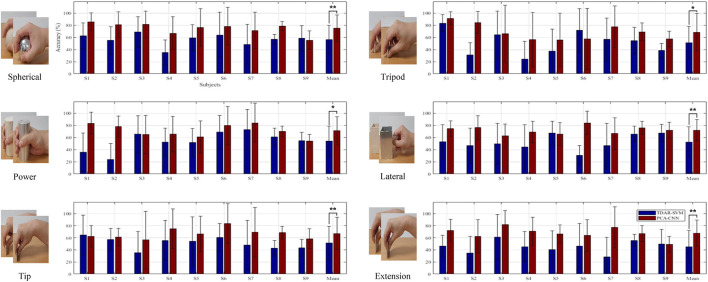
Averaged results of classifying different weights (light/heavy) within same grasping types (spherical, tripod, power, lateral, tip, and extension grip). S in *x*-axis indicates each subject (* and ** means *p*-value < 0.05 and 0.01, respectively).

Figure 7 presents the 10-fold cross-validation results of the TDAR–SVM and the PCA–CNN for classifying the different weighted (light/heavy) objects within each reaching-to-grasping task (spherical, tripod, power, lateral, tip, and extension). In Figure 7, the EMG data from the six channels were used for calculating each accuracy. The averaged accuracies of the TDAR–SVM and PCA–CNN (binary classification) were calculated as 56.5 ± 23.4% and 75.0 ± 22.2% for the spherical, 51.3 ± 27.0% and 68.4 ± 30.7% for the tripod, 53.9 ± 25.0% and 71.2 ± 22.9% for the power, 52.4 ± 25.0% and 71.8 ± 17.5% for the lateral, 51.3 ± 27.8% and 66.8 ± 27.1% for the tip, and 45.3 ± 27.1% and 67.8 ± 21.6% for the extension. In all subjects, the PCA–CNN method showed a substantial-high accuracy, 15% more than the TDAR–SVM method, as the average accuracies (2-classes) were 51.8 ± 13.2% and 70.1 ± 9.8%, respectively.

Furthermore, the main factor of ANOVA was showed *p* < 0.01 in each classification accuracies of all reaching-to-grasping tasks. This means that statistically significant differences existed between the TDAR–SVM and PCA–CNN for classifying the reaching-to-grasping tasks. Based on these experimental results, we can confirm that the PCA–CNN can improve the accuracy of classifying reaching-to-grasping tasks for light and heavy objects.

## Discussion

In this study, the PCA–CNN method was applied to classify reaching-to-grasping tasks using the EMG signals from only the upper arm and upper body without wrist muscles. To the authors’ best knowledge, reaching-to-grasping tasks were classified using the EMG signals from only the upper arm and upper body for considering the upper limb amputees who may not have or have weak below-elbow muscle activities for the first time. In the previous study classifying reaching-to-grasping tasks (Martelloni et al., 2008), three healthy subjects performed reaching-to-grasping three different objects (a key, a tennis ball, and a tin) placed in three directions (30° in the contralateral hemisphere, 0 and 30° in the lateral hemisphere). For grasping these objects, each subject performed a lateral, a spherical, and a palmar grip, respectively, Martelloni et al. (2008). The MAV features were extracted from acquired EMG signals, and the SVM was used as a classifier (Martelloni et al., 2008). Consequently, each reaching-to-grasping for three objects was classified with accuracies of 55.6% (a key), 66.7% (a ball), and 59.3% (a tin) (Martelloni et al., 2008). The EMG was acquired from seven electrodes at the deltoideus pars anterior, trapezius pars ascendens, pectoralis major pars clavicularis, triceps brachii caput longum, biceps brachii caput longum, extensor carpi radialis, and flexor carpi radialis (Martelloni et al., 2008). The accuracies were derived, including the EMG signals from two electrodes at the forearm (Martelloni et al., 2008). In our experimental results (Figure 5), the averaged classification result was 69.4 ± 11.4% (PCA–CNN with six channels) despite using the EMG signals from only the upper arm and upper body. Therefore, the PCA–CNN method can be suitable for classifying reaching-to-grasping tasks based on the EMG signals from the upper arm and upper body.

Overall, the PCA–CNN method showed better performance for classifying reaching-to-grasping than the TDAR–SVM method. However, the PCA–CNN did not show better performance in all subjects. Interestingly, in the *trans*-radial and wrist amputees (S8 and S9), the previous TDAR–SVM method showed better performances. It is possible that due to more variation in EMG signals in the amputees compared with healthy individuals, more data from the amputees may be needed to train the CNN classifier (especially the tip and extension). Following the previous study about deep learning (Vedaldi and Lenc, 2015), the traditional machine learning approaches can be shown better performance for lesser amounts of input data (Alom et al., 2019). As the amount of data increases beyond a certain number, then the deep learning approach can increase the classification accuracy (Alom et al., 2019). Based on our approach and methods, additional experiments with more trials and a large population may be needed to further investigate the effectiveness of the PCA–CNN method on the classification of reaching-to-grasping in amputees. Furthermore, in the case of the S3, classification accuracy was lower than other subjects (Figures 5, 6). It can be interpreted that the TDAR–SVM and PCA–CNN methods were not suitable to classify for reaching-to-grasping tasks of the S3. Therefore, various approaches will be performed as further research. First of all, as the possible combination of feature extraction and classifier, the TDAR–CNN and PCA–SVM will be applied to classifying the reaching-to-grasping tasks. In the previous study (Zhai et al., 2016), the PCA and the root mean square, as one of TDAR, were compared. In the study, the PCA showed a better performance in classifying hand movements (Zhai et al., 2016) among only healthy subjects; thus, indeed, more studies are required to validate the effectiveness of PCA. Furthermore, more advanced machine learning techniques, such as the recurrent neural networks, could be applied and compared for our further study for considering a case like the S3.

In our study, the PCA–CNN and the TDAR–SVM methods classified the tip and extension grip with low performances (Figure 6). As seen in Figure 3, the tip and extension grip showed similar hand posture while performing the tasks. More advanced machine learning techniques may be required to improve accuracy in classifying the cases of tip and extension. Therefore, we have a plan to analyze the EMG signals using advanced techniques such as the generative adversarial networks and conduct additional experiments for performance comparison.

To the authors’ best knowledge, reaching-to-grasping with the light and heavy objects were classified using the EMG signals from only the upper arm and upper body for the first time. Therefore, it is difficult to compare our averaged accuracy with previous studies (Martelloni et al., 2008). However, a comparison of classification accuracies between the TDAR–SVM, which was widely used, and the PCA–CNN method could be made. The classification accuracies of the PCA–CNN method shown much higher, 15% over, than the TDAR–SVM method (Figure 7). Following these results, the PCA–CNN can classify the differences mentioned earlier in the amplitudes of EMG signals with higher accuracies than the TDAR–SVM method. In future work, additional experiments with an object with more than three weights will be implemented to make it clearer regarding the possibility of classifying the weights.

## Conclusion

In this article, we investigated the possibility of classifying reaching-to-grasping tasks using the EMG signals from the upper arm and upper body. Furthermore, the TDAR–SVM and the PCA–CNN methods were compared for decoding the reaching-to-grasping tasks using only upper-limb EMG signals. Our experimental results showed that the PCA–CNN method could classify not only the six reaching-to-grasping but also different weighted (light and heavy) objects with better performance than the TDAR–SVM method. However, in the cases of *trans*-radial and wrist amputees, additional experiments with more trials and other types of amputee patients are required for the validation of the effectiveness of the PCA–CNN method.

Furthermore, the classification accuracy depends on various subject-specific factors such as concentration level, tiredness, etc. Therefore, these factors also require additional investigation with a large population to determine how they could affect the EMG-based upper-limb movements.

In further investigation, to make it clearer in regards to the effectiveness of the PCA–CNN method, the real-time myoelectric interfaces will be constructed, and external robotic devices, such as a robotic arm or hand, control experiments will be implemented.

## Data Availability Statement

The original contributions presented in the study are included in the article/supplementary material, further inquiries can be directed to the corresponding author/s.

## Ethics Statement

The studies involving human participants were reviewed and approved by the Institutional Review Board of the Korea Institute of Science and Technology. The patients/participants provided their written informed consent to participate in this study.

## Author Contributions

K-TK and SL contributed to the study’s conception and design. SP and T-HL performed the experimental environment configuration and data collection. K-TK and SL performed data analysis and wrote the first draft of the manuscript. All authors considerably revised the manuscript and approved the final manuscript.

## Conflict of Interest

The authors declare that the research was conducted in the absence of any commercial or financial relationships that could be construed as a potential conflict of interest.

## Publisher’s Note

All claims expressed in this article are solely those of the authors and do not necessarily represent those of their affiliated organizations, or those of the publisher, the editors and the reviewers. Any product that may be evaluated in this article, or claim that may be made by its manufacturer, is not guaranteed or endorsed by the publisher.

## References

[B1] Alom M. Z. Taha T. M. Yakopcic C. Westberg S. Sidike P. Nasrin M. S. (2019). A state-of-the-art survey on deep learning theory and architectures. *Electronics* 8:292. 10.3390/electronics8030292

[B2] Al-Timemy A. H. Khushaba R. N. Bugmann G. Escudero J. (2015). Improving the performance against force variation of EMG controlled multifunctional upper-limb prostheses for transradial amputees. *IEEE Trans. Neural Syst. Rehabil. Eng.* 24 650–661. 10.1109/tnsre.2015.2445634 26111399

[B3] Ameri A. Kamavuako E. N. Scheme E. J. Englehart K. B. Parker P. A. (2014). Support vector regression for improved real-time, simultaneous myoelectric control. *IEEE Trans. Neural Syst. Rehabil. Eng.* 22 1198–1209. 24846649 10.1109/TNSRE.2014.2323576

[B4] Atzori M. Cognolato M. Müller H. (2016). Deep learning with convolutional neural networks applied to electromyography data: a resource for the classification of movements for prosthetic hands. *Front. Neurorobot.* 10:9. 10.3389/fnbot.2016.00009 27656140 PMC5013051

[B5] Batzianoulis I. El-Khoury S. Pirondini E. Coscia M. Micera S. Billard A. (2017). EMG-based decoding of grasp gestures in reaching-to-grasping motions. *Robot. Autonom. Syst.* 91 59–70. 10.1016/j.robot.2016.12.014

[B6] Brochier T. Spinks R. L. Umilta M. A. Lemon R. N. (2004). Patterns of muscle activity underlying object-specific grasp by the macaque monkey. *J. Neurophysiol.* 92 1770–1782. 10.1152/jn.00976.2003 15163676

[B7] Castellini C. Fiorilla A. E. Sandini G. (2009). Multi-subject/daily-life activity EMG-based control of mechanical hands. *J. Neuroeng. Rehabil.* 6 1–11. 10.1007/978-1-84800-063-6_1 19919710 PMC2784470

[B8] Chowdhury R. H. Reaz M. B. Ali M. A. B. M. Bakar A. A. Chellappan K. Chang T. G. (2013). Surface electromyography signal processing and classification techniques. *Sensors* 13 12431–12466. 10.3390/s130912431 24048337 PMC3821366

[B9] Chu J.-U. Moon I. Lee Y.-J. Kim S.-K. Mun M.-S. (2007). A supervised feature-projection-based real-time EMG pattern recognition for multifunction myoelectric hand control. *IEEE/ASME Trans. Mechatron.* 12 282–290. 10.1109/tmech.2007.897262

[B10] Dalley S. A. Varol H. A. Goldfarb M. (2011). A method for the control of multigrasp myoelectric prosthetic hands. *IEEE Trans. Neural Syst. Rehabil. Eng.* 20 58–67. 10.1109/tnsre.2011.2175488 22180515 PMC3372414

[B11] Englehart K. Hudgins B. (2003). A robust, real-time control scheme for multifunction myoelectric control. *IEEE Trans. Biomed. Eng.* 50 848–854. 10.1109/tbme.2003.813539 12848352

[B12] Fligge N. Urbanek H. Van Der Smagt P. (2013). Relation between object properties and EMG during reaching to grasp. *J. Electromyogr. Kinesiol.* 23 402–410. 10.1016/j.jelekin.2012.10.010 23207412

[B13] Fougner A. Stavdahl O. Kyberd P. J. Losier Y. G. Parker P. A. (2012). Control of upper limb prostheses: terminology and proportional myoelectric control—a review. *IEEE Trans. Neural Syst. Rehabil. Eng.* 20 663–677. 10.1109/tnsre.2012.2196711 22665514

[B14] Fukuda O. Tsuji T. Kaneko M. Otsuka A. (2003). A human-assisting manipulator teleoperated by EMG signals and arm motions. *IEEE Trans. Robot. Automat.* 19 210–222. 10.1109/tra.2003.808873

[B15] Gaudet G. Raison M. Achiche S. (2018). Classification of upper limb phantom movements in transhumeral amputees using electromyographic and kinematic features. *Eng. Appl. Artif. Intell.* 68 153–164. 10.1016/j.engappai.2017.10.017

[B16] Hargrove L. Losier Y. Lock B. Englehart K. Hudgins B. (2007). A real-time pattern recognition based myoelectric control usability study implemented in a virtual environment. *Annu. Int. Conf. IEEE Eng. Med. Biol. Soc.* 2007 4842–4845. 18003090 10.1109/IEMBS.2007.4353424

[B17] Ison M. Artemiadis P. (2014). The role of muscle synergies in myoelectric control: trends and challenges for simultaneous multifunction control. *J. Neural Eng.* 11:051001. 10.1088/1741-2560/11/5/051001 25188509

[B18] Jarrassé N. Nicol C. Touillet A. Richer F. Martinet N. Paysant J. (2016). Classification of phantom finger, hand, wrist, and elbow voluntary gestures in transhumeral amputees with sEMG. *IEEE Trans. Neural Syst. Rehabil. Eng.* 25 71–80. 10.1109/tnsre.2016.2563222 27164596

[B19] Jitaree S. Phukpattaranont P. (2019). Force classification using surface electromyography from various object lengths and wrist postures. *Signal Image Video Process.* 13 1183–1190. 10.1007/s11760-019-01462-z

[B20] Jones L. A. Lederman S. J. (2006). *Human Hand Function.* Oxford: Oxford university press.

[B21] Kamakura N. Matsuo M. Ishii H. Mitsuboshi F. Miura Y. (1980). Patterns of static prehension in normal hands. *Am. J. Occup. Ther.* 34 437–445. 10.5014/ajot.34.7.437 6446851

[B22] Khan S. M. Khan A. A. Farooq O. (2021). Pattern recognition of EMG signals for low level grip force classification. *Biomed. Phys. Eng. Express* 7:065012. 10.1088/2057-1976/ac2354 34474400

[B23] Kim J.-H. Bießmann F. Lee S.-W. (2014). Decoding three-dimensional trajectory of executed and imagined arm movements from electroencephalogram signals. *IEEE Trans. Neural Syst. Rehabil. Eng.* 23 867–876. 10.1109/tnsre.2014.2375879 25474811

[B24] Kim K.-T. Guan C. Lee S.-W. (2019). A subject-transfer framework based on single-trial EMG analysis using convolutional neural networks. *IEEE Trans. Neural Syst. Rehabil. Eng.* 28 94–103. 10.1109/tnsre.2019.2946625 31613773

[B25] Kyberd P. J. Murgia A. Gasson M. Tjerks T. Metcalf C. Chappell P. H. (2009). Case studies to demonstrate the range of applications of the southampton hand assessment procedure. *Br. J. Occup. Ther.* 72 212–218. 10.1177/030802260907200506

[B26] MacKenzie C. L. Iberall T. (1994). *The Grasping Hand.* Amsterdam: Elsevier.

[B27] Marotta J. J. Medendorp W. Crawford J. (2003). Kinematic rules for upper and lower arm contributions to grasp orientation. *J. Neurophysiol.* 90 3816–3827. 10.1152/jn.00418.2003 12930815

[B28] Martelloni C. Carpaneto J. Micera S. (2008). “Classification of upper arm EMG signals during object-specific grasp,” in *Proceedings of the Annual International Conference of the IEEE Engineering in Medicine and Biology Society. IEEE Engineering in Medicine and Biology Society. Annual Conference*, (Vancouver, BC). 10.1109/IEMBS.2008.4650351 19163854

[B29] Marteniuk R. Mackenzie C. Jeannerod M. Athenes S. Dugas C. (1987). Constraints on human arm movement trajectories. *Can. J. Psychol.* 41:365. 10.1037/h0084157 3502905

[B30] Mcsp I. C. B. Dipcot J. A. (2003). A comparison of dominant and non-dominant hand function in both right-and left-handed individuals using the Southampton Hand Assessment Procedure (SHAP). *Br. J. Hand Ther.* 8 4–10. 10.1177/175899830300800101

[B31] Napier J. R. (1956). The prehensile movements of the human hand. *J. Bone Joint Surg. Br. Vol.* 38 902–913. 10.1302/0301-620x.38b4.902 13376678

[B32] Ouyang G. Zhu X. Ju Z. Liu H. (2013). Dynamical characteristics of surface EMG signals of hand grasps via recurrence plot. *IEEE J. Biomed. Health Inform.* 18 257–265. 10.1109/jbhi.2013.2261311 24403424

[B33] Park K.-H. Suk H.-I. Lee S.-W. (2015). Position-independent decoding of movement intention for proportional myoelectric interfaces. *IEEE Trans. Neural Syst. Rehabil. Eng.* 24 928–939. 10.1109/tnsre.2015.2481461 26415203

[B34] Peerdeman B. Boere D. Witteveen H. Hermens H. Stramigioli S. Rietman H. (2011). Myoelectric forearm prostheses: state of the art from a user-centered perspective. *J. Rehabil. Res. Dev.* 48 719–737. 10.1682/jrrd.2010.08.0161 21938658

[B35] Phinyomark A. Quaine F. Charbonnier S. Serviere C. Tarpin-Bernard F. Laurillau Y. (2013). EMG feature evaluation for improving myoelectric pattern recognition robustness. *Expert Syst. Appl.* 40 4832–4840. 10.1016/j.eswa.2013.02.023

[B36] Santello M. Flanders M. Soechting J. F. (1998). Postural hand synergies for tool use. *J. Neurosci.* 18 10105–10115. 10.1523/jneurosci.18-23-10105.1998 9822764 PMC6793309

[B37] Saponas T. S. Tan D. S. Morris D. Turner J. Landay J. A. (2010). “Making muscle-computer interfaces more practical,” in *Proceedings of the SIGCHI Conference on Human Factors in Computing Systems*, (Atlanta, GA), 851–854.

[B38] Sapsanis C. Georgoulas G. Tzes A. Lymberopoulos D. (2013). “Improving EMG based classification of basic hand movements using EMD,” in *Proceedings of the 2013 35th Annual International Conference of the IEEE Engineering in Medicine and Biology Society (EMBC)*, (Osaka: IEEE). 10.1109/EMBC.2013.6610858 24111045

[B39] Scheme E. Englehart K. (2011). Electromyogram pattern recognition for control of powered upper-limb prostheses: state of the art and challenges for clinical use. *J. Rehabil. Res. Dev.* 48 643–659. 10.1682/jrrd.2010.09.0177 21938652

[B40] Shenoy P. Miller K. J. Crawford B. Rao R. P. (2008). Online electromyographic control of a robotic prosthesis. *IEEE Trans. Biomed. Eng.* 55 1128–1135. 10.1109/tbme.2007.909536 18334405

[B41] Smith R. J. Tenore F. Huberdeau D. Etienne-Cummings R. Thakor N. V. (2008). “Continuous decoding of finger position from surface EMG signals for the control of powered prostheses,” in *Proceedings of the 2008 30th Annual International Conference of the IEEE Engineering in Medicine and Biology Society*, (Montpellier: IEEE), 197–200. 10.1109/IEMBS.2008.4649124 19162627

[B42] Vedaldi A. Lenc K. (2015). “Matconvnet: convolutional neural networks for matlab,” in *Proceedings of the 23rd ACM international conference on Multimedia*, Brisbane.

[B43] Wolf M. T. Assad C. Vernacchia M. T. Fromm J. Jethani H. L. (2013). “Gesture-based robot control with variable autonomy from the JPL BioSleeve,” in *Proceedings of the 2013 IEEE International Conference on Robotics and Automation*, (Karlsruhe: IEEE).

[B44] Young A. J. Smith L. H. Rouse E. J. Hargrove L. J. (2012). Classification of simultaneous movements using surface EMG pattern recognition. *IEEE Trans. Biomed. Eng.* 60 1250–1258. 10.1109/tbme.2012.2232293 23247839 PMC4208826

[B45] Zardoshti-Kermani M. Wheeler B. C. Badie K. Hashemi R. M. (1995). EMG feature evaluation for movement control of upper extremity prostheses. *IEEE Trans. Rehabil. Eng.* 3 324–333. 10.1109/86.481972

[B46] Zhai X. Jelfs B. Chan R. H. Tin C. (2016). “Short latency hand movement classification based on surface EMG spectrogram with PCA,” in *Proceedings of the 2016 38th Annual International Conference of the IEEE Engineering in Medicine and Biology Society (EMBC)*, (Orlando, FL: IEEE). 10.1109/EMBC.2016.7590706 28268343

[B47] Zhai X. Jelfs B. Chan R. H. Tin C. (2017). Self-recalibrating surface EMG pattern recognition for neuroprosthesis control based on convolutional neural network. *Front. Neurosci.* 11:379. 10.3389/fnins.2017.00379 28744189 PMC5504564

